# Functional and embedding feature analysis for pan-cancer classification

**DOI:** 10.3389/fonc.2022.979336

**Published:** 2022-09-29

**Authors:** Jian Lu, JiaRui Li, Jingxin Ren, Shijian Ding, Zhenbing Zeng, Tao Huang, Yu-Dong Cai

**Affiliations:** ^1^ Department of Mathematics, School of Sciences, Shanghai University, Shanghai, China; ^2^ CAS Key Laboratory of Computational Biology, Bio-Med Big Data Center, Shanghai Institute of Nutrition and Health, University of Chinese Academy of Sciences, Chinese Academy of Science, Shanghai, China; ^3^ Advanced Research Computing, University of British Columbia, Vancouver, BC, Canada; ^4^ School of Life Sciences, Shanghai University, Shanghai, China; ^5^ CAS Key Laboratory of Tissue Microenvironment and Tumor, Shanghai Institute of Nutrition and Health, University of Chinese Academy of Sciences, Chinese Academy of Sciences, Shanghai, China

**Keywords:** pan-cancer, cancer mutation, enrichment, embedding, feature selection, rule learning

## Abstract

With the increasing number of people suffering from cancer, this illness has become a major health problem worldwide. Exploring the biological functions and signaling pathways of carcinogenesis is essential for cancer detection and research. In this study, a mutation dataset for eleven cancer types was first obtained from a web-based resource called cBioPortal for Cancer Genomics, followed by extracting 21,049 features from three aspects: relationship to GO and KEGG (enrichment features), mutated genes learned by word2vec (text features), and protein-protein interaction network analyzed by node2vec (network features). Irrelevant features were then excluded using the Boruta feature filtering method, and the retained relevant features were ranked by four feature selection methods (least absolute shrinkage and selection operator, minimum redundancy maximum relevance, Monte Carlo feature selection and light gradient boosting machine) to generate four feature-ranked lists. Incremental feature selection was used to determine the optimal number of features based on these feature lists to build the optimal classifiers and derive interpretable classification rules. The results of four feature-ranking methods were integrated to identify key functional pathways, such as olfactory transduction (hsa04740) and colorectal cancer (hsa05210), and the roles of these functional pathways in cancers were discussed in reference to literature. Overall, this machine learning-based study revealed the altered biological functions of cancers and provided a reference for the mechanisms of different cancers.

## 1 Introduction

Cancer is one of the most common causes of death in human beings. According to World Health Organization (WHO), about 10 million patients died because of cancer in 2020. Early cancer diagnosis significantly improves the survival, but more than half of patients with cancer have been diagnosed in advanced stages (1). The average 5-year survival rate after surgery in the early stage is 91%, which is higher than the 26% survival rate in the late stage (2).

The identification of tumor type and tissue origin is of paramount importance for cancer treatment. Most cancer types are diagnosed *via* invasive biopsy; however, non-invasive early detection is lacking (3). Circulating tumor DNA (ctDNA) could be a potential biomarker for early cancer diagnosis (4). Despite the multiple challenges in developing non-invasive liquid biopsy based on ctDNA in blood plasma, such as the limited materials of cancer DNA in blood plasma to achieve a high sensitivity (5), enormous efforts and progresses have been made in the past decades. Studies on identification methods for tumor tissue of origin mainly focused on characterizing and utilizing tumor-specific DNA methylation, gene expression profiling, and genomic alteration (6–8). Machine learning methods, especially deep learning models, have been developed and widely used to identify tumor tissue of origin (9). In our previous study, we developed a bioinformatics pipeline based on machine learning algorithms to identify the tissue of origin in five tumors according to the enrichment of gene ontology (GO) terms and Kyoto Encyclopedia of Genes and Genomes (KEGG) using the mutated genes (10); the approach was proven to be of high efficacy and robustness. However, the limitation of previous methods in analyzing small datasets restricted our previous analysis with only five cancer types.

In this study, we applied machine learning algorithms to investigate a large mutation data, which involved eleven cancer types. Each sample was represented by three feature types: (1) relationship to GO terms and KEGG pathways; (2) word embeddings of mutated genes; (3) network embeddings of mutated genes. Several machine learning algorithms were applied to such dataset. First, the irrelevant features were excluded by Boruta feature selection. Then, remaining features were deeply analyzed by four different feature selection methods, resulting in four feature-ranked lists. In the next step, each feature list is subjected to incremental feature selection (IFS) (11) combined with the different classification algorithms to determine the optimal number of features and build the optimal classifiers. Some essential features were identified by each feature selection method and those identified by multiple methods were deemed to be more important. Features related to GO terms and KEGG pathways were analyzed. Furthermore, this study also reported several classification rules, indicating different patterns on various cancer types. From the results yielded by four feature selection methods, they were quite different, suggesting that the four methods are complement with each other. Incorporating multiple methods in the pipeline can help us achieve a more comprehensive result.

## 2 Materials and methods

### 2.1 Data sources

Mutation data with eleven cancer types were acquired from the cBioPortal for Cancer Genomics (http://cbio.mskcc.org/cancergenomics/pancan_tcga /) (12, 13). This dataset mainly includes bladder urothelial carcinoma (BLCA), breast invasive carcinoma (BRCA), colon adenocarcinoma/rectum adenocarcinoma esophageal carcinoma (COADREAD), glioblastoma multiforme (GBM), head and neck squamous cell carcinoma (HNSC), kidney renal clear cell carcinoma (KIRC), acute myeloid leukemia (LAML), lung adenocarcinoma (LUAD), lung squamous cell carcinoma (LUSC), ovarian serous cystadenocarcinoma (OV), and uterine corpus Endometrial Carcinoma (UCEC). A total of 3478 samples were obtained, and the sample size for each cancer type is listed in **Table 1**. This cancer mutation dataset was then used in the next step of the analysis.

**Table 1:** Number of samples under different cancer types.

Cancer type	Sample size
BLCA	100
BRCA	513
COADREAD	499
GBM	276
HNSC	306
KIRC	473
LAML	201
LUAD	230
LUSC	177
OV	456
UCEC	247

### 2.2 Feature representation

In this work, three approaches were utilized to encode the feature vectors to extract relevant information from each cancer sample in the mutant dataset: GO and KEGG enrichment theory, word2vec, and node2vec. Accordingly, three feature types were generated from each sample, namely, enrichment, text and network features, respectively. A total of 21,049 features were created, with 20,293 enrichment features derived from GO and KEGG, 256 text features yielded by word2vec, and 500 network features generated by node2vec. A detailed description of these features is presented below.

#### 2.2.1 Enrichment features derived from GO and KEGG

GO terms and KEGG pathways give crucial functional information for gene characterization in biology study and the discovery of underlying biological mechanisms. The data obtained could be helpful for further research when GO terms and KEGG pathways are used for feature encoding. As a commonly used approach in quantifying the overlap between the gene set and GO terms or KEGG pathways, the GO and KEGG enrichment theory (14) were used to measure the impact of alterations in biological functions among patients with cancer.

For a specific cancer individual *p* and a GO term *GO_j_*, *G_GO_* represents the gene set that is annotated by *GO_j_*, and *G_p_* represents the variant gene set for individual *p*. The relationship between *p* and *GO_j_* is defined as the hypergeometric test p-values of *G_p_* and *G_GO_*, called GO enrichment score, which can be computed by


(1) $$Score_{GO}(p,\;GO_{j})=-\log_{10}(\sum_{k=m}^{n}\frac{\left(\begin{array}{l} M\\ k \end{array}\right)\left(\begin{array}{l} N-M\\ n-k \end{array}\right)}{\left(\begin{array}{l} N\\ n \end{array}\right)}),$$

where *N* and *M* indicate the total number of human genes and the number of genes in *G_GO_*, respectively; *n* denotes the number of mutant genes in *G_p_*, and *m* represents the number of genes both in *G_p_* and *G_GO_*. According to the high enrichment score, the mutation in patient *p* has a deep functional impact on the GO term *GO_j_*.

Similarly, for the KEGG pathway, the enrichment score for a cancer individual *p* and a KEGG pathway *K_j_* can be calculated as follows, called KEGG enrichment score,


(2) $$Score_{KEGG}(p,K_{j})\;=-log_{10}(\sum_{k=m}^{n}\frac{\left(\begin{array}{l} M\\ k \end{array}\right)\left(\begin{array}{l} N-M\\ n-k \end{array}\right)}{\left(\begin{array}{l} N\\ n \end{array}\right)}),$$

where *N* and *n* are defined as shown in **Eq. 1**, and *M* and *m* indicate the number of genes in pathway *K_j_* and the number of genes both in *G_p_* and *K_j_*. A total of 20,293 GO terms and KEGG pathways were adopted in this study, with the enrichment scores between patients with cancer and these functional terms serving as the feature values. Each patient with cancer is represented by 20,293 enrichment scores, which can be used for subsequent feature analysis. For convenience, such features were called enrichment features. These features were calculated by our in-house program.

#### 2.2.2 Text features generated by Word2vec

Word2vec is a natural language processing model that uses unsupervised learning to learn word associations from a text corpus (15). It obtains the word embedding vectors by training two-layer neural networks to reconstruct linguistic contexts of words, making the semantic and syntactic similar words close in distance in a specific space. Words are embedded in a continuous vector space, with close vectors for similar words. The training algorithms of word2vec are mainly CBOW or Skip-gram. Here, the word2vec algorithm in Gensim (https://github.com/RaRe-Technologies/gensim) was adopted. It took the name of each gene as a word and the genes presenting in each sample as sentences. The second class of features was the average of the vectors corresponding to the genes under each sample. In summary, word2vec program with default parameters was used to produce a 256-dimensional feature vector for each sample based on gene names. For convenience, these features were called text features.

#### 2.2.3 Network features generated by Node2vec

The gene interaction network provides information on the features of gene interactions. In this study, gene names were inputted into a gene network based on the PPI network in STRING (16). Each node in this network represents a gene, each edge denotes the interaction between two genes. Evidently, each edge indicates a PPI. To reflect different strengths of PPIs, edges are assigned the confidence scores of their corresponding PPIs. Thus, this gene interaction network is a weighted version. The feature vector of each gene is obtained using node2vec (17).

The node2vec algorithm can be regarded as a generalized version of Skip-gram, which can process network data. It first generates several paths starting from each node in the network. Each path is extended from the current endpoint to one of its neighbors in a well-defined way. After a predefined number of paths have been generated, they are fed into the word2vec with Skip-gram, where nodes in paths are termed as words and paths are considered as sentences, to yield the feature vector of each node. The node2vec program was retrieved from https://snap.stanford.edu/node2vec/. Default parameters were used.

The gene interaction network mentioned above was fed into the node2vec program, assigning a vector feature for each node (gene). The feature vector of each sample was further constructed from the feature vectors of genes and was defined as the mean vector of the feature vectors of genes related to the sample. In this study, a 500-dimensional feature vector was generated for each of the samples from the gene interaction network. For convenience, these features were called network features.

### 2.3 Feature selection methods

#### 2.3.1 Boruta feature filtering

A total of 21,049-dimensional feature vectors were obtained after feature encoding. Directly employing these features for analysis would require massive computation. Therefore, non-essential features were eliminated from the dataset using Boruta feature selection (18). In each iteration round, Boruta compares the importance of the original feature to that of the shadow feature with random forest (RF) classifier. If the original feature is statistically more important than the shadow features, then the original feature is deemed important. If the original feature is statistically less essential than the shadow feature, then the original feature is considered unimportant. After Boruta analysis, the important features were retained for the next step of feature ranking, and the computational efficiency was improved.

In this work, the Boruta program from https://github.com/scikit-learn-contrib/boruta_py was used and executed with default parameters.

#### 2.3.2 Least absolute shrinkage and selection operator

Lasso (19) is a regression model that uses L1 regularization technology. The overfitting problem is reduced by adding a high penalty to parameters with high coefficients and great prediction errors, thus reducing the number of parameters and lowering the feature dimension because some feature coefficients are reduced to 0 and eliminated from the model. As a result, Lasso is frequently used for the selection of features that are prioritized by importance according to their coefficients. The Lasso package, obtained from Scikit-learn (20), was applied on the features selected by Boruta. Its default parameters were adopted. The obtained feature list was called Lasso feature list.

#### 2.3.3 Minimum redundancy maximum relevance

mRMR (21) is a feature selection method that has been widely applied in biology. Its main goal is to maximize the correlation between features and categorical variables while minimizing feature-to-feature redundancy. Mutual information between individual features and category variables is used to determine the correlation between features and categories, and mutual information between features and features is used to calculate the redundancy. A ranked feature list can be obtained after feature selection using mRMR. The mRMR program was derived from http://home.penglab.com/proj/mRMR/, and it was executed with default parameters. The list yielded by mRMR was called mRMR feature list.

#### 2.3.4 Monte Carlo feature selection

MCFS (22) is used to identify the essential features in the dataset for a particular classification problem. The method resamples the original dataset *c* times, separates it into *c* pairs of training and test sets, randomly selects *m* features from all features for *s* times to build a decision tree (DT), and generates *s* DTs each time. Finally, the entire procedure yields c ×*s* DTs.

The relative importance (RI) is computed for each feature based on these DTs. Features are sorted in descending order of RI values to produce a ranked feature list. Here, MCFS was implemented using the dmLab software provided by Draminski (22) with parameters *u* and *v* set to 1, which can be obtained at http://www.ipi-pan.eu/staff/m.draminski/mcfs.html. Features with RI scores equal to 0 from the calculation results were deleted in the next analysis. The list yielded by MCFS was termed as MCFS feature list.

#### 2.3.5 Light gradient boosting machine

LightGBM (23) is a fast gradient boosting DT implementation that recurrently fits a new DT by using the negative gradient of the loss function of the current DT as the approximate value of the residual. This approach saves computer resources by employing two strategies called gradient-based one-side sampling and exclusive feature bundling. Given that LightGBM is based on a tree model, the importance of a feature can be quantified by the number of times the feature is involved in building the DTs. In this study, a python version of the LightGBM program with default parameters, which can be downloaded from https://lightgbm.readthedocs.io/en/latest/, was used to rank features selected by Boruta. This list was called LightGBM feature list.

### 2.4 Incremental feature selection

Features selected by Boruta were sorted in descending order of importance using the Lasso, mRMR, MCFS, and lightGBM algorithms. However, the features in each feature list that were critical to the classification of cancer types were not determined. Therefore, IFS (11) was used to detect the optimal number of features in each ranked list and build the optimal classifiers.

Given a feature list, IFS produces a series of feature subsets depending on a specified interval step initially. For example, when the interval is 5, the first feature subset includes the top 5 features in the list, and the second feature subset includes the top 10 features. All possible feature subsets can be generated when the interval was set as 1. The sample data including each of these feature subsets are then applied to train a classifier with a given classification algorithm (e.g., DTs (24), random forest (RF) (25), and support vector machine (SVM) (26)). Such classifier is tested by 10-fold cross-validation (Kohavi, 1995). When training the classifier, we adopted Synthetic Minority Oversampling Technique (SMOTE) (27) to balance the sample sizes of different cancer types in this study. Ultimately, all classifiers built by the succession of feature subsets were compared using a performance metric to determine the optimal number of features and the consequent optimal classifier.

### 2.5 Synthetic minority oversampling technique

In this study, the sample sizes for the eleven cancer types were markedly unequal as indicated in **Table 1**. The obtained results are frequently unsatisfactory when the classifier is built by directly utilizing an unbalanced sample dataset. Hence, overcoming the categorization difficulty provided by uneven data has become a machine learning challenge. SMOTE is a synthetic sampling strategy in which new samples for a minority class are generated using any randomly selected sample and its nearest neighbors (27). In this study, SMOTE was utilized in the imblearn module (with default parameters) to synthesize new samples for minority cancer types and generate an equal number of cancer samples in each type in the training set.

### 2.6 Classification algorithms

In IFS method, one classification algorithm was necessary. To fully test each feature subset, three classic classification algorithms: DT (24), RF (25), and SVM (26), were employed in this study. These classification algorithms have been applied to tackle various medical or biological problems (28–36).

#### 2.6.1 Support vector machine

SVM is one of the most classic classification algorithms. The main idea is to determine a hyperplane by learning the distribution of samples in different classes. Generally, such hyperplane in the original feature space is difficult to obtain. SVM adopts the kernel trick to translate samples to a high-dimensional feature space. In this case, such hyperplane is easy to discover. The class of a test sample is determined according to the side of the hyperplane it belongs to.

#### 2.6.2 Random forest

RF is also a classic classification algorithm, which is quite different from SVM. In fact, it is an ensemble algorithm consisting of several DTs. Each DT is built on a new dataset, in which samples were randomly selected, with replacement, from the original dataset. And such new dataset has same number of samples in the original dataset. Furthermore, each DT is constructed based on randomly selected features. The predicted results of RF are determined by the majority voting on the results yielded by all DTs.

#### 2.6.3 Decision tree

Above two classification algorithms are generally deemed to be powerful. However, their decision principles are quite complicated, which is impossible for us to understand. This is a great block for us to learn new knowledge from a large dataset. For this study, we cannot extract mutation patterns on different cancer types only based on RF and SVM. In view of this, DT was also used in this study, which is deemed to be a type of white-box algorithm. It employs a tree structure and contains leaf nodes and branch nodes. The branch nodes are in charge of classifying samples, whereas the leaf nodes are responsible for determining classes. Besides the tree representation, a DT can also be represented by a set of IF-THEN rules. Each rule is obtained by a path from root node to one leaf node. These rules make the classification procedures completely open, providing opportunities for us to understand different patterns on various cancer types.

In this work, the corresponding packages that implement above SVM, RF and DT, in scikit-learn (20) were employed. Each package was performed with default parameters.

### 2.7 Performance measurement

In the IFS, the classifiers were trained using training samples consisting of the feature subsets. The performance of the classifiers was then evaluated using 10-fold cross-validation (37). The commonly used main model metrics for each class are accuracy (recall), precision and *F*1 score (38–41). Here, *F*_1_ score was used as the main metric to measure the performance of the classifier on one class, which can be calculated as follows:


(3) $$F1\;score=\frac{2\times precious\times recall}{precious+recall}$$

As above F1 score only measures the performance of the classifier on one class. F1 scores on all classes can be integrated to give an overall evaluation on the classifier. There are two ways to integrate these scores. The first way is to calculate the mean of all F1 scores. Such obtained measurement is called macro F1. The second way further considers the class sizes, the weighted mean of all F1 scores is computed, which is termed as weighted F1. As the sizes of different cancers are quite different, weighted F1 was more proper than macro F1 to fully evaluate the overall performance of classifiers. Thus, it was selected as the key measurement in this study.

Besides, the overall accuracy (ACC) and Matthew correlation coefficients (MCC) (42, 43) were also employed. ACC is a generally measurement, which indicates the proportion of correctly predicted samples. MCC is much more complex. However, it is deemed as a balanced measurement even if the sizes of classes are quite different. To compute MCC, two matrices *X* and *Y* should be constructed in advance, where *X* stores the true class of each sample and *Y* includes the predicted class of each sample. Then, the MCC can be computed by


(4) $$MCC=\frac{\mathrm{cov}(X,Y)}{\sqrt{\mathrm{cov}(X,X)\mathrm{cov}(Y,Y)}},$$

where cov(Y,X) stands for the covariance of two matrices.

## 3 Results

**Figure 1:**
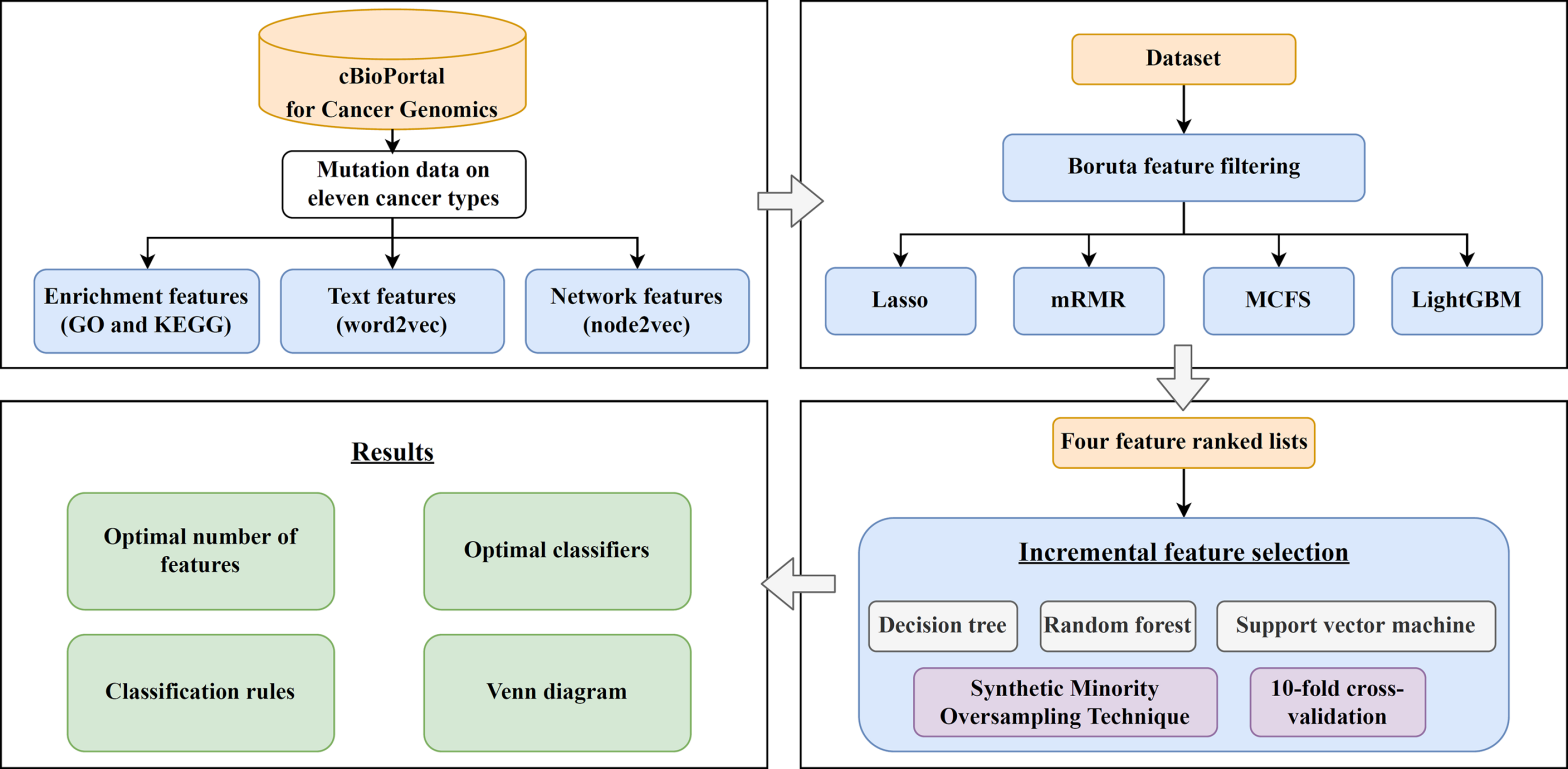
Computational framework of this study. First, the cancer samples obtained from the cBioPortal for Cancer Genomics database are represented by three feature types, derived by GO and KEGG enrichment, word2vec, and node2vec. Then, the Boruta feature filtering is adopted to exclude irrelevant features and retained features are ranked by Lasso, mRMR, MCFS, and LightGBM methods in four feature-ranked lists. These feature lists are subjected to incremental feature selection combined with classification algorithms to determine the optimal number of features, build optimal classifiers, and extract important classification rules. Furthermore, the Venn diagram analysis is conducted on the key features identified by different feature selection methods.

In this study, we first downloaded a mutation dataset containing 3478 cancer samples from the cBioPortal for Cancer Genomics database, which included eleven cancer types. Three feature types (enrichment, text and network features) were generated to represent each cancer sample. The Boruta feature filtering method was used to remove irrelevant features and selected features were further analyzed by Lasso, mRMR, MCFS, and LightGBM methods, respectively, to produce four feature-ranked lists. Each feature list was subjected to IFS combined with classification algorithms and model evaluation measurements to determine the optimal number of features, build the optimal classifiers, and extract the important classification rules. The entire analysis pipeline is shown in **Figure 1**. This section details the obtained results.

### 3.1 Results of feature selection methods

**Figure 2:**
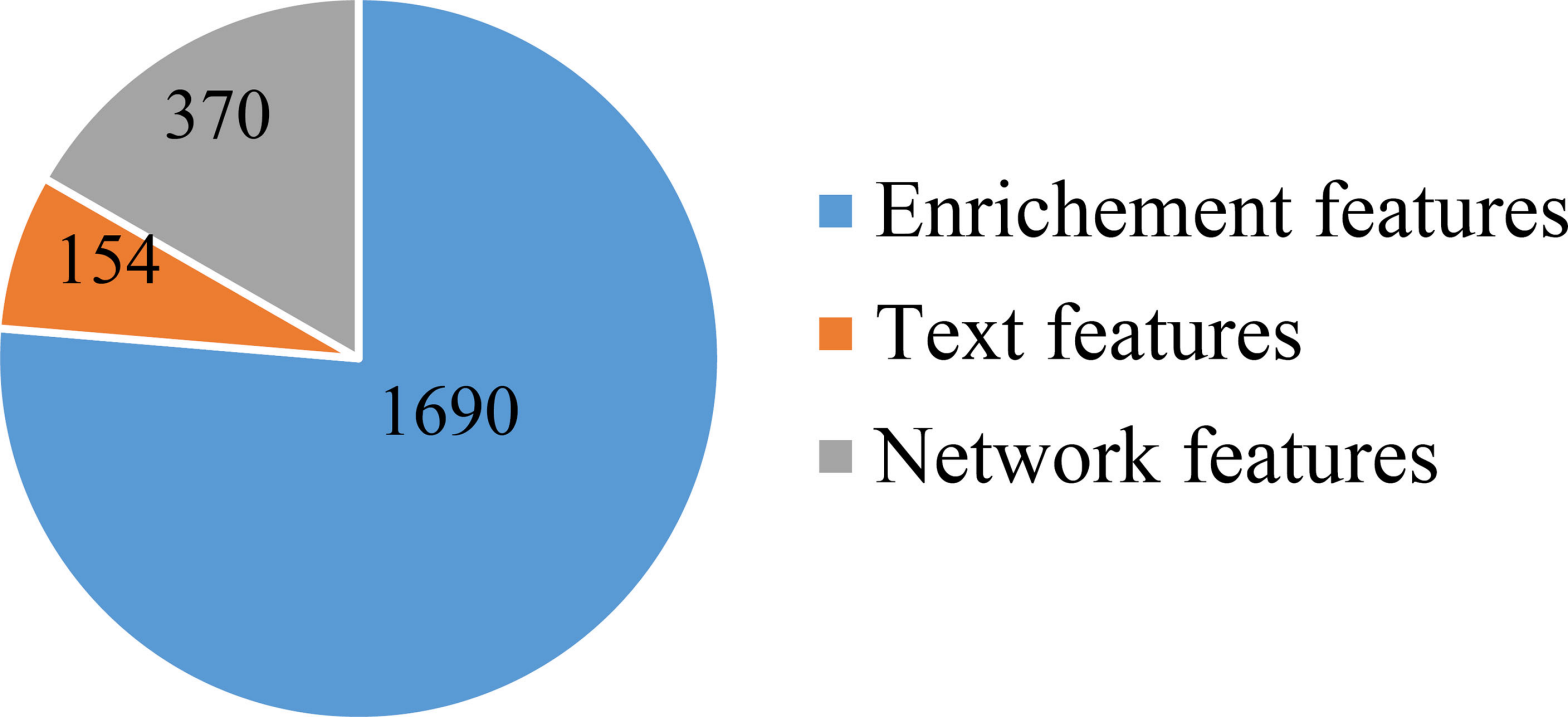
Pie chart to show the distribution of features selected by Boruta on three feature types. Enrichment features are most, followed by network and text features.

First, a large dataset containing 3,478 samples and 21,049 features was generated. To filter key informative features from these features, the Boruta feature filtering method was applied to such dataset. 18,835 features were excluded and 2,214 important features were retained, which are provided in **Supplementary Table S1**. Among the selected 2,214 features, enrichment features were most, followed by network and text features. The numbers of selected features on three types are shown in **Figure 2**. Enrichment features were important to classify samples into different cancer types. However, considering the fact that the original enrichment features were much more than other two feature types, such result was reasonable. Furthermore, the selected enrichment features only occupied 8.33% of all enrichment features, and such proportions for text and network features were 60.16% and 74.00%, respectively. It was indicated that text and network features also provided key contributions on the classification of cancer samples.

In the next step, a refined dataset with 3,478 samples and 2,214 selected features was produced. Four feature selection methods (Lasso, mRMR, MCFS, and LightGBM) were executed on such dataset to analyze the importance of the 2,214 features. Four feature lists: Lasso, mRMR, MCFS and LightGBM feature lists, were obtained. These lists are also provided in **Supplementary Table S1**.

### 3.2 Results of IFS method on different feature lists

We obtained four feature-ranked lists but were still unable to determine the features in each list that could effectively distinguish cancer types. Therefore, we employed the IFS combined with classification algorithms to determine the optimal results. For each list, IFS first generated a series of feature subsets with interval 5, on which the DT, RF, and SVM classifiers were constructed. We then used 10-fold cross-validation for evaluation with weighted F1 as the key performance metric. The results of the IFS on different feature selection methods are provided in **Supplementary Table S2**.

**Figure 3:**
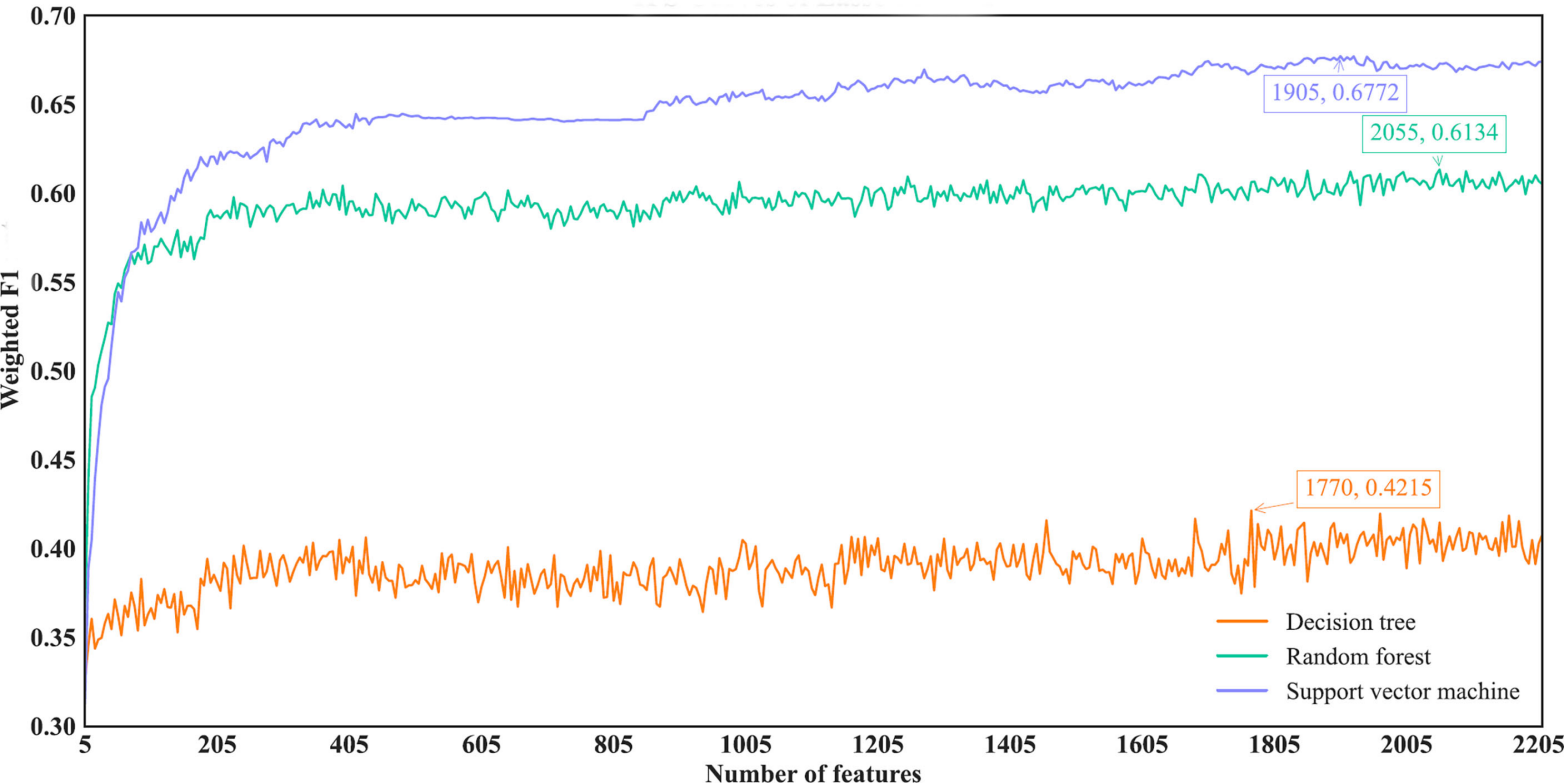
IFS curves of different classification algorithms on the Lasso feature list. Three classification algorithms provided highest weighted F1 values of 0.4215, 0.6134 and 0.6772, respectively, based on top 1770, 2055 and 1905, respectively, features in the list.

**Figure 4:**
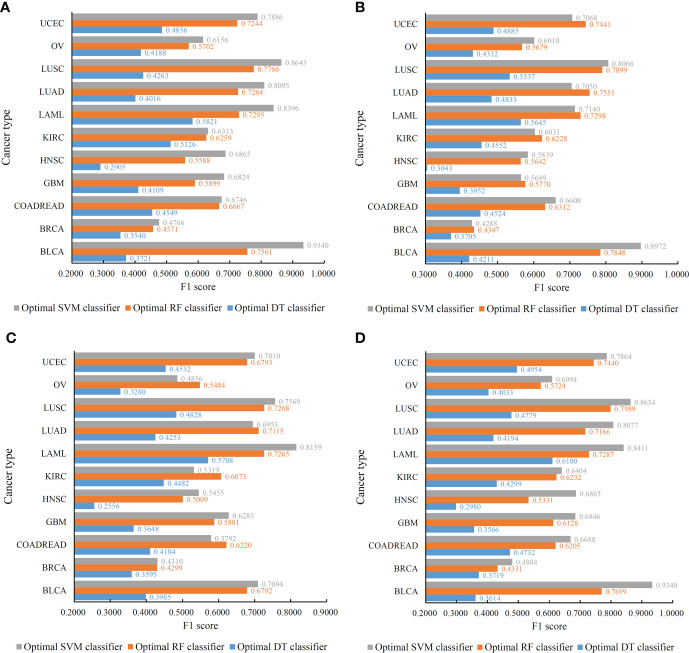
Performance of the optimal classifiers on eleven cancer types for different feature lists. **(A)** Lasso feature list; **(B)** mRMR feature list; **(C)** MCFS feature list; **(D)** LightGBM feature list.

For the IFS results on the Lasso feature list, an IFS curve was plotted for each classification algorithm, as shown in **Figure 3**, where weighted F1 was set as Y-axis and number of features was set as X-axis. It can be observed that the highest weighted F1 values for DT, RF and SVM were 0.4215, 0.6134 and 0.6772, respectively. These values were obtained by using top 1770, 2055 and 1905, respectively, features in the list, which constituted the optimal feature subsets for three classification algorithms, respectively. Furthermore, the optimal DT, RF and SVM classifiers were built using the corresponding optimal feature subsets. The values of ACC, MCC and Macro F1 yielded by these optimal classifiers are listed in **Table 2**. Evidently, the optimal SVM classifier provided the highest performance. The performance of the optimal classifiers on eleven cancer types are illustrated in **Figure 4A**, from which we can see that the optimal SVM classifier provided the best performance on all cancer types. This further confirmed the superiority of the optimal SVM classifier.

**Table 2:** Detailed performance of the optimal classifiers for different feature selection methods and classification algorithms.

Feature selection method + classification algorithm	Number of features	ACC	MCC	Macro F1	Weighted *F*_1_
Lasso + DT	1770	0.4218	0.3574	0.4270	0.4215
Lasso + RF	2055	0.6236	0.5844	0.6503	0.6134
Lasso + SVM	1905	0.6811	0.6443	0.7275	0.6772
mRMR + DT	490	0.4373	0.3748	0.4454	0.4347
mRMR + RF	1505	0.6268	0.5880	0.6547	0.6170
mRMR + SVM	1810	0.6271	0.5873	0.6611	0.6200
MCFS + DT	460	0.3982	0.3325	0.4088	0.3966
MCFS + RF	385	0.6024	0.5615	0.6200	0.5917
MCFS + SVM	550	0.5871	0.5401	0.6254	0.5823
LightGBM + DT	1880	0.4278	0.3645	0.4281	0.4273
LightGBM + RF	315	0.6288	0.5893	0.6529	0.6218
LightGBM + SVM	2015	0.6803	0.6430	0.7275	0.6771

**Figure 5:**
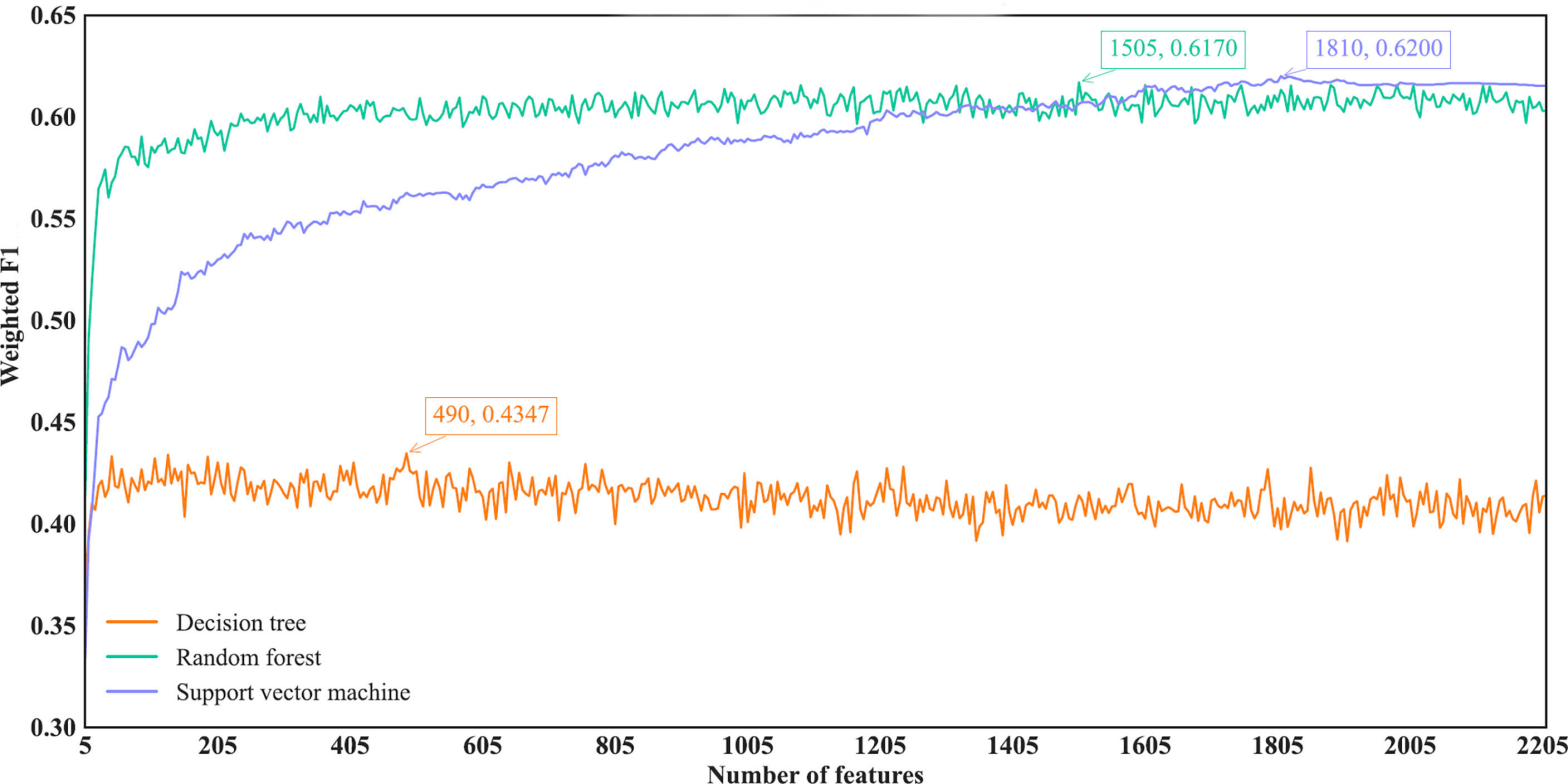
IFS curves of different classification algorithms on the mRMR feature list. Three classification algorithms provided highest weighted F1 values of 0.4347, 0.6170 and 0.6200, respectively, based on top 490, 1505 and 1810, respectively, features in the list.

With regard to the IFS results on the mRMR feature list, three IFS curves were also plotted, as illustrated in **Figure 5**. DT, RF and SVM provided the highest weighted F1 of 0.4347, 0.6170 and 0.6200, respectively. Top 490, 1505 and 1810, respectively, features in the mRMR feature list were used to generate such performance. On these features, the optimal DT, RF and SVM classifiers were built. Their additional performance measurements are listed in **Table 2** and **Figure 4B**. The optimal SVM classifier still gave the highest performance. However, its superiority to the optimal RF classifier was not very evident. The optimal DT/RF classifier gave almost the equal performance of the optimal DT/RF classifier on Lasso feature list, but the performance of the optimal SVM classifier was evidently declined compared with that of the optimal SVM classifier on the Lasso feature list.

**Figure 6:**
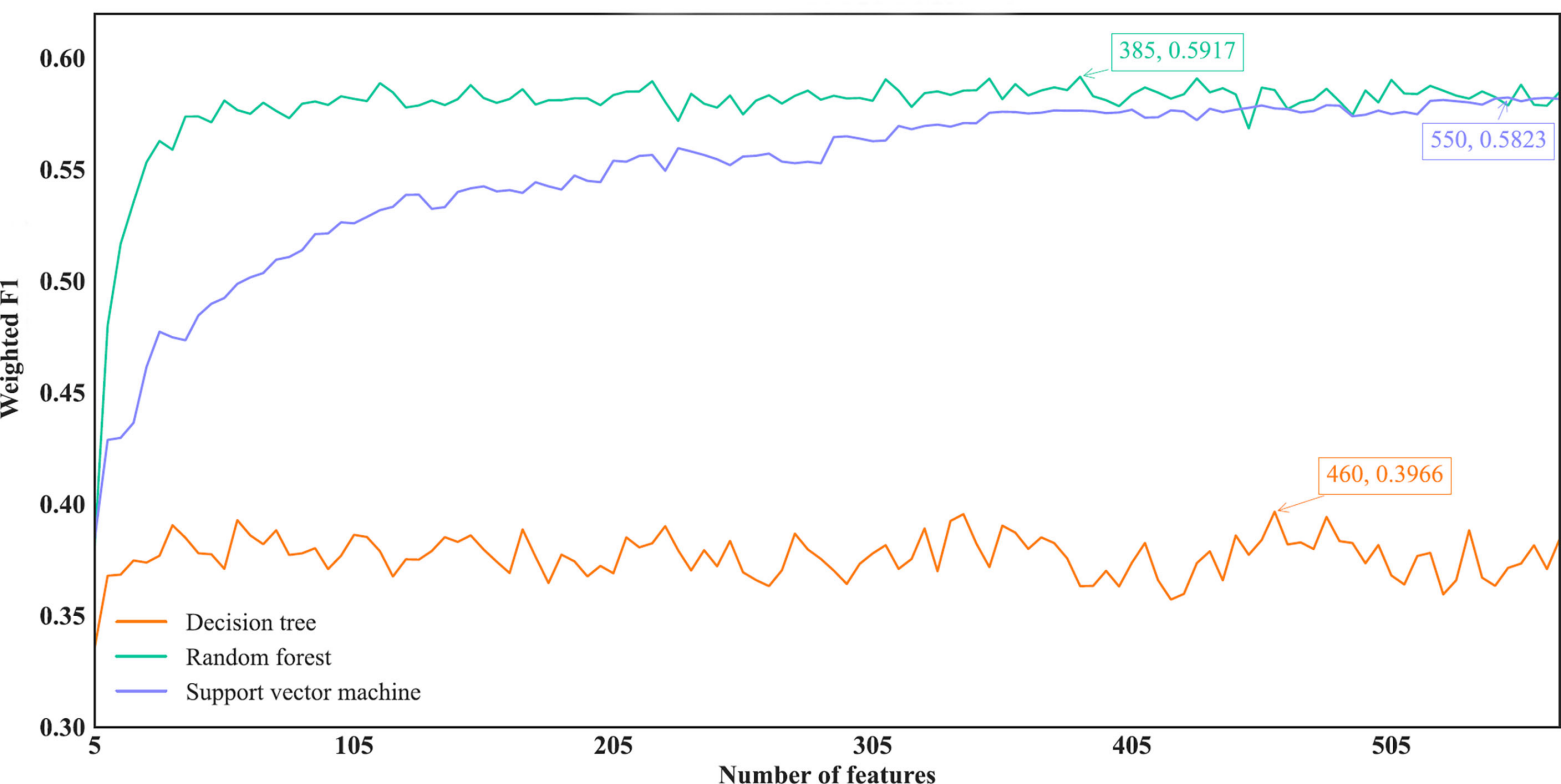
IFS curves of different classification algorithms on the MCFS feature list. Three classification algorithms provided highest weighted F1 values of 0.3966, 0.5917 and 0.5823, respectively, based on top 460, 385 and 550, respectively, features in the list.

For the IFS results on the MCFS feature list, we also plotted an IFS curve for each classification algorithm to clearly show its performance on different feature subsets, as illustrated in **Figure 6**. When top 460, 385 and 550, respectively, features in the list were used, DT, RF and SVM provided the highest weighted F1 values of 0.3966, 0.5917 and 0.5823, respectively. Accordingly, the optimal DT, RF and SVM classifiers were built with these optimal features. Their ACC, MCC and Macro F1 are listed in **Table 2** and their performance on eleven cancer types is shown in **Figure 4C**. The optimal RF and SVM classifiers were almost at the same level. Relatively speaking, the optimal RF classifier was slightly better than the optimal SVM classifier. These three optimal classifiers gave lower performance than above optimal classifiers using the same classification algorithm.

**Figure 7:**
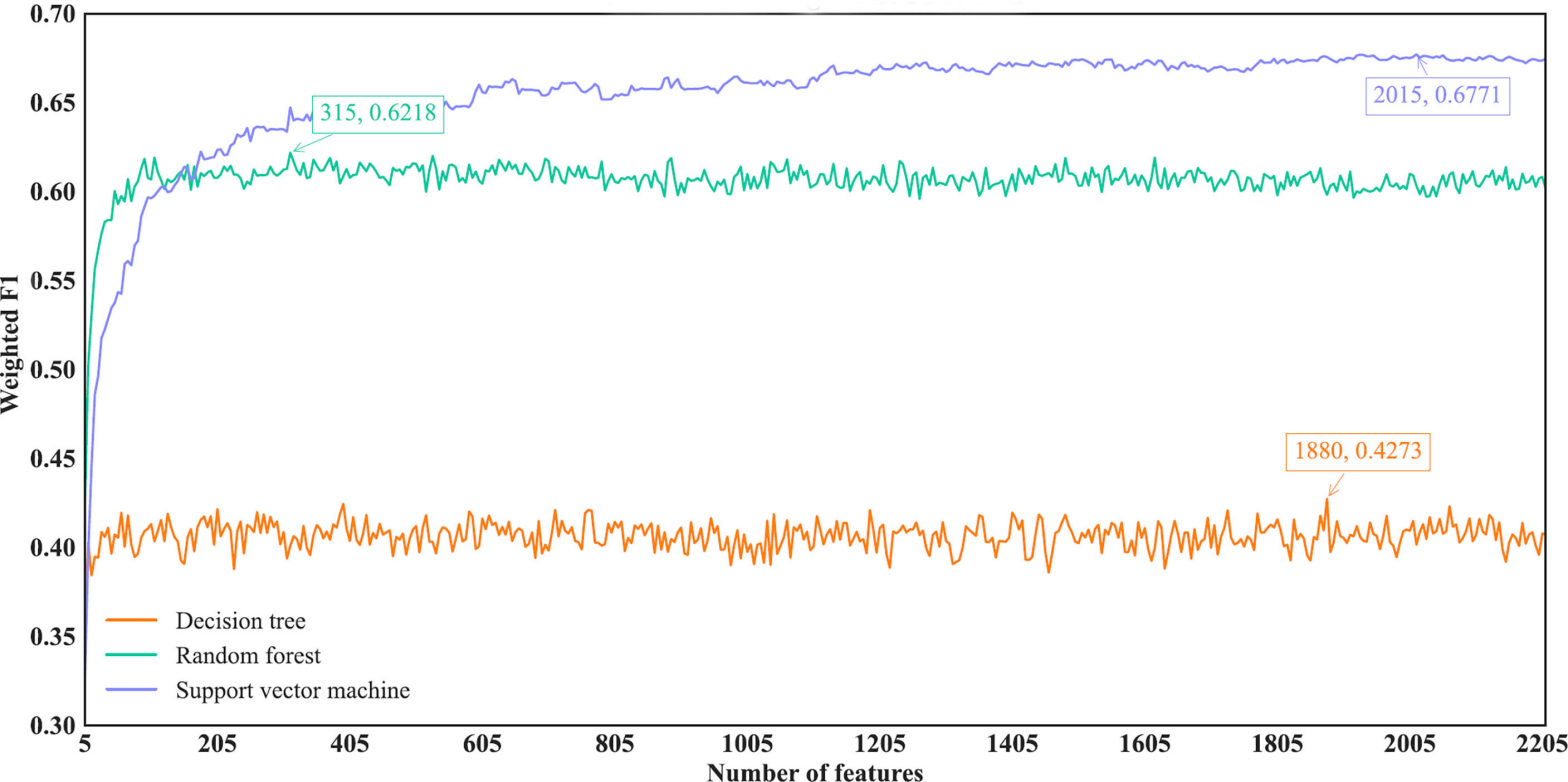
IFS curves of different classification algorithms on the LightGBM feature list. Three classification algorithms provided highest weighted F1 values of 0.4273, 0.6218 and 0.6771, respectively, based on top 1880, 315 and 2015, respectively, features in the list.

As for the IFS results on the LightGBM feature list, similar investigation was conducted. **Figure 7** shows the three IFS curves of three classification algorithms. It can be observed that DT, RF and SVM provided the highest weighted F1 values of 0.4273, 0.6218 and 0.6771, respectively, when top 1880, 315 and 2015, respectively, features in the list were adopted. These features were used to build the optimal DT, RF and SVM classifiers. Other overall measurements of these optimal classifiers are listed in **Table 2**. And their performance on all cancer types is shown in **Figure 4D**. Evidently, the optimal SVM classifier was better than other two classifiers. The performance of these classifiers is quite similar to that of the optimal classifiers on the Lasso feature list.

Among above optimal classifiers, the optimal SVM classifiers on the Lasso and LightGBM feature lists were evidently better than other classifiers. They can be efficient tools to classify cancer samples into different types.

### 3.3 Investigation on key features

**Figure 8:**
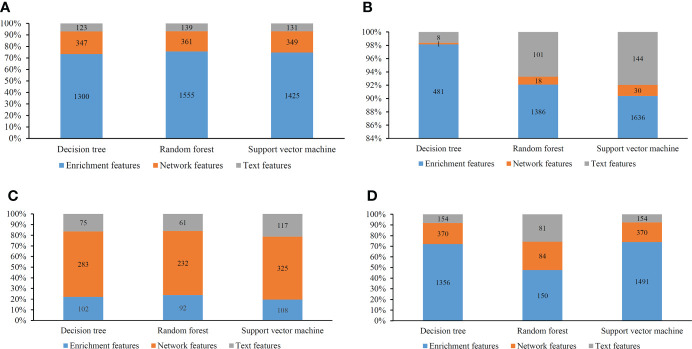
Distribution of the optimal features for different classification algorithms and feature lists. **(A)** Lasso feature list; **(B)** mRMR feature list; **(C)** MCFS feature list; **(D)** LightGBM feature list.

Several optimal classifiers were built in Section 3.2. Features used in these classifiers were deemed to be related to cancer classification. Here, we investigated the distribution of these features on three feature types. The distribution is shown in **Figure 8**. Except the results on MCFS feature list, enrichment features always occupied most. Network features were most for the results on MCFS feature list. Based on different feature selection methods, some common features can be extracted, whereas some exclusive features can also be discovered by a certain feature selection method. Integrating the results derived from different feature selection methods can give a full overview on cancer classification.

**Figure 9:**
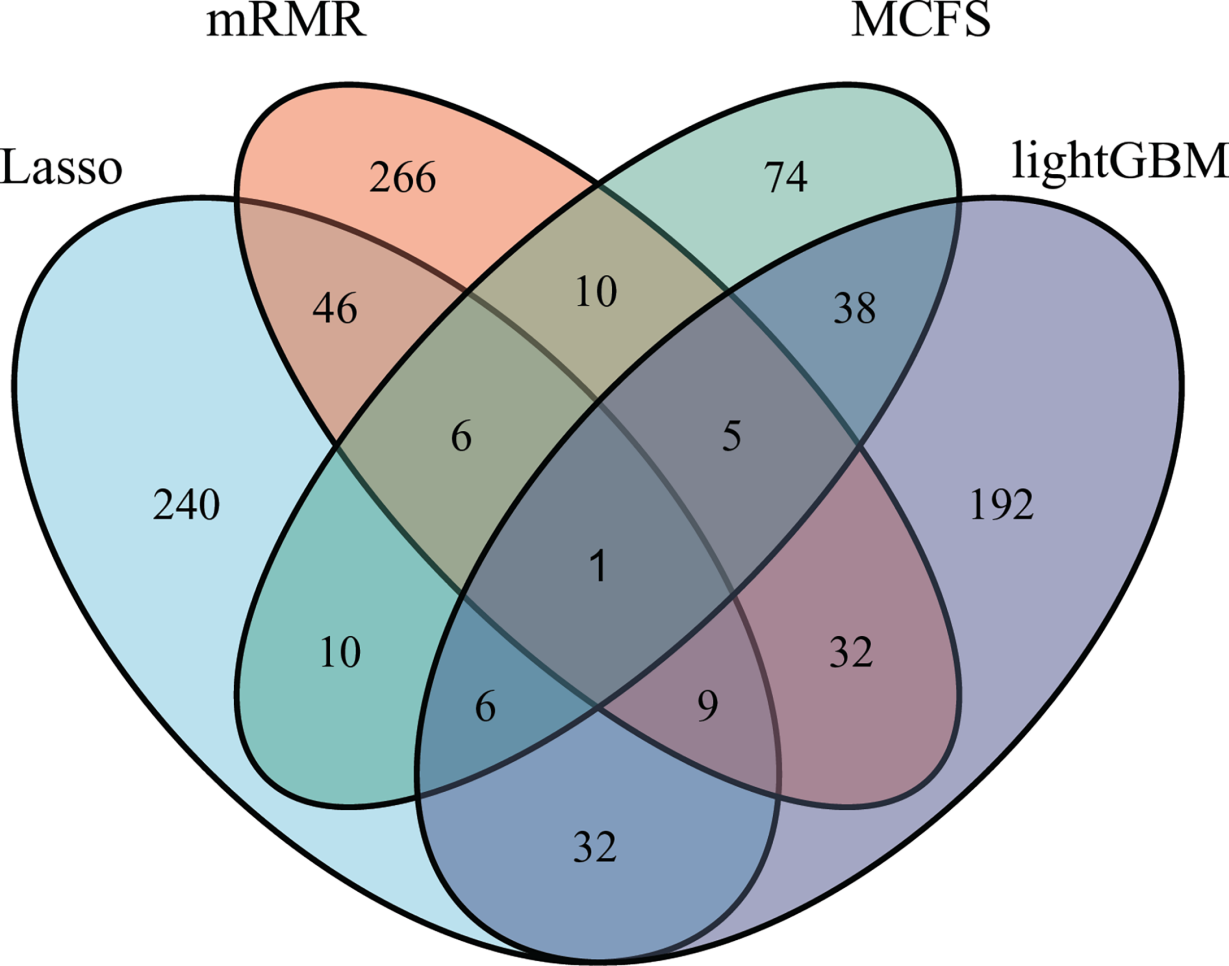
Venn diagram to show the intersection of key features identified by different feature selection methods. Lasso, mRMR, MCFS and LightGBM indicate the feature subsets identified by the feature selection methods with the same names.

As mentioned in Section 3.2, the optimal SVM classifier was generally the best among all optimal classifiers on a certain feature list. However, these classifiers were of low efficiency due to the large number of features used. In view of this, we carefully checked the IFS results with SVM, trying to finding out a SVM classifier with high performance but with less features. Finally, the SVM classifiers using top 350 features in the Lasso feature list, top 375 features in the mRMR feature list, top 150 features in the MCFS feature list and top 315 features in the LightGBM feature list were picked up. For convenience, these classifiers were called feasible SVM classifiers. Their performance is listed in **Table 3**. It can be observed that their performance is slightly lower than the corresponding optimal SVM classifiers. However, they were more efficient than the optimal classifiers as much less features were involved. Since the optimal features except those used in the feasible classifiers can provide limited improvement, features used in the feasible classifiers were evidently more important than the rest optimal features. Further investigation on these features was helpful to uncover the essential differences of various cancer types. Thus, four feature sets consisting of features in four feasible SVM classifiers were set up and a Venn diagram was plotted, as shown in **Figure 9**. Detailed intersections are listed in **Supplementary Table S3**. The results showed that the four feature sets intersected in one important feature (hsa04740), with 26 intersections in three feature sets. These features were deemed to be highly related to cancer classification. Among these 27 (1 + 26) features, 20 were enrichment features (occupied 74%), three were network features and four were text features. The biological implications of these features for cancer classification would be presented in Section 4.1.

**Table 3:** Performance of the feasible SVM classifiers for different feature selection methods.

Feature selection method	Number of features	ACC	MCC	Macro F1	Weighted F1
Lasso	350	0.6449	0.6038	0.6903	0.6401
mRMR	375	0.5604	0.5150	0.5756	0.5527
MCFS	150	0.5472	0.4957	0.5874	0.5416
LightGBM	315	0.6521	0.6124	0.6887	0.6472

### 3.4 Classification rules derived from the optimal DT classifiers

Although SVM generally achieved the best performance in the above tasks, it is a black-box algorithm that is difficult to interpret in a biological sense. Meanwhile, DT has a low predictive power but can provide easily understandable decision rules because of its IF-THEN rule architecture, which simplifies the discussion on the biological implications of the features. In view of this, DT was used to conduct some additional investigations.

**Figure 10:**
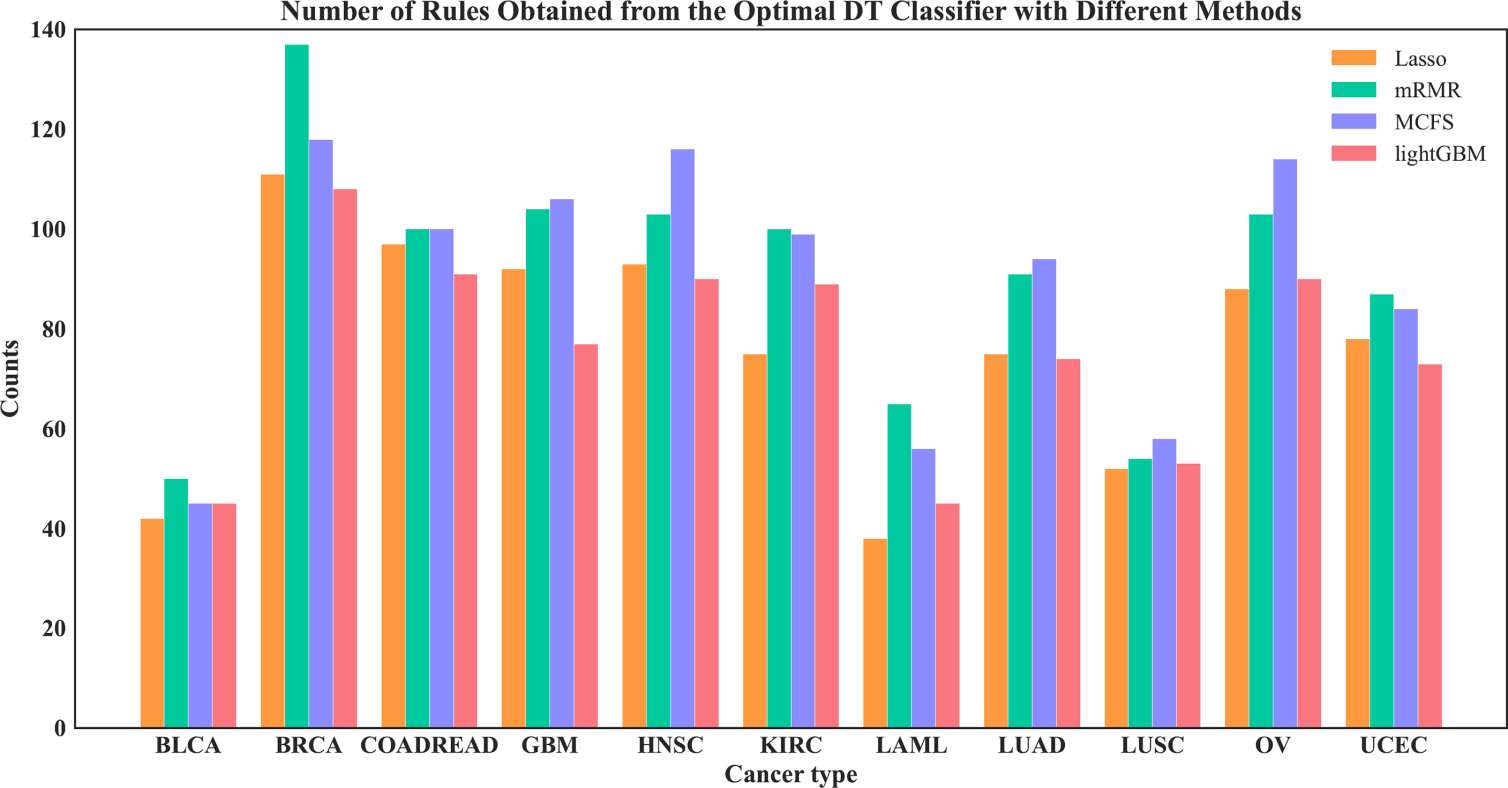
Number of rules under each cancer type obtained by the four optimal DT classifiers on four feature lists.

On each feature list, the number of the optimal features for DT had been determined *via* IFS method. Based on these features, DT was applied to all cancer samples and a tree was learned. A set of classification rules was extracted from such tree, which is provided in **Supplementary Table S4**. The number of rules for each cancer type under different feature selection methods is shown in **Figure 10**. The results showed that BRCA and HNSC were given plenty of classification rules. The biological importance of these rules would be discussed in Section 4.2.

## 4 Discussion

We compared the optimal feature sets identified by the four feature selection methods and found that these methods generated different results (**Figure 9**). A total of 195 optimal features were identified by two or more feature selection methods, and 772 features were method-specific. The features shared by multiple methods may play key roles in cancer-type-specific development. For example, the feature hsa04550, which is the KEGG pathway of “Signaling pathways regulating pluripotency of stem cells - Homo sapiens (human)” was shared by three methods. Tumorigenesis and the generation of induced pluripotent stem cells (iPSCs) are highly similar processes, and iPSCs from different cell types are led by different reprogramming processes (44). Only one feature was shared by all four methods, which is hsa04740, the KEGG pathway “Olfactory transduction - Homo sapiens (human)”. Previous studies found that the 301 olfactory receptor genes showed different expression patterns in 968 cancer cell lines derived from different cancer types (45); this finding indicated the specific roles of this pathway in different cancers.

### 4.1 Clustering of optimal GO terms features indicated the functional groups in categorizing the cancer types

Given the abundance of algorithm-specific features, we applied Revigo to cluster the GO terms to assess the relevance of these features in cancer categorization (46). This relevance infers the distance between two GO terms according to the pair-wise semantic similarity. We highlighted the GO terms representing the clusters and ranked the top by four algorithms by displaying their descriptions.

#### 4.1.1 Analysis of biological process

**Figure 11:**
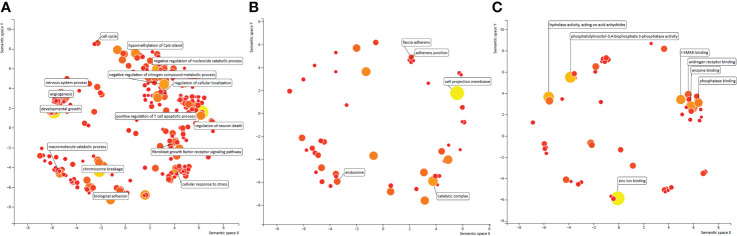
**(A)** GO term clustering of Biological Process identified by only one of the four algorithms. The distance between two GO terms were inferred based on pair-wise semantic similarities. **(B)** GO term clustering of Cellular Component identified by only one of the four algorithms. The distance between two GO terms were inferred based on pair-wise semantic similarities. **(C)** GO term clustering of Molecular Function identified by only one of the four algorithms. The distance between two GO terms were inferred based on pair-wise semantic similarities.

Our literature review confirmed the relevance of these GO terms and clusters to cancer type classification (**Figure 11A**). For example, a cluster was enriched with GO terms involved in T cell responses. T-cell apoptosis could be triggered by up-regulating FAS/FASL system in cancer cells. The mutations in FAS and FASL genes reduce the risks of certain types of cancer but not the others, indicating that T cell apoptosis behaves differently in different cancers. IN the present study, cellular responses, especially immune responses involved in T cells, were one of the most critical function groups in distinguishing cancer types. The polymorphisms of the fundamental immunosuppressive cytokine, cytotoxic T-lymphocyte antigen-4 (CTLA4, CD152), which terminates the T-cell response and prohibits T-cell activation, are associated with the risk of breast and cervical cancers (47). This finding proved again the relevance of these GO term clusters to cancer types. In addition, the GO terms involved in fibroblast growth factor receptor (FGFR) signaling pathway are clustered because FGFRs are recurrently altered in many human cancers. The prevalence of the mutations in this gene depends on the cancer type (48). The other three GO terms clusters include chromosome damage or rearrangement, cellular or tissue development, and regulations of biological processes, including epigenetic modifications. These biological processes have specific signatures in different cancers: different tumors with different origin of cell types are underlined by cancer-type-specific tumorigenesis processes because of the diverse characteristics of different cell types. Studies using the Pan-Cancer Analysis of Whole Genome (PCAWG) and The Cancer Genome Atlas (TCGA) data identified chromoanagenesis landscape in different cancers (49), implying the different distributions of mutation types. Moreover, different tumor development mechanisms are caused by aneuploidy, a context-dependent, cancer-type-specific oncogenic event (50).

#### 4.1.2 Analysis of molecular function

In contrast to biological process, we saw only 1 cluster enriched in the other two categories of GO terms, molecular function and cellular component. This cluster was found in the GO term category of cellular function and was enriched by several protein-protein binding functions, such as I-SMAD binding. The nuclear accumulation of active Smad complexes transduces the transforming growth factor beta-superfamily signals from transmembrane receptors into the nucleus. Genetic and epigenetic changes, such as DNA mutations, methylation, and miRNA expression, contribute to the transcriptional activity of TGF-β signaling in all cancer types (51). Previous studies identified the mutation hotspots in SMAD and inhibitors, indicating that the different alterations of Smad and the binding proteins play an important role in different cancer types by regulating TGF-β signaling through various ways.

#### 4.1.3 Analysis of cellular component

In addition to the only cluster enriched by GO terms involved in protein-protein binding, the other GO terms in Cellular Component and Molecular Function showed less similarity with each other (**Figures 11B**, **11C**). However, the top ranked GO terms were well recognized as highly cancer type specific. For example, the top GO term in Cellular Component was cell projection membrane, which is a cell protrusion that is involved in many biological functions, such as cancer cell invasion, cell motility, and cytokinesis. Glypicans play a role in cellular and tissue development, morphogenesis, and cell motility and show differential expression in different cancer types by behaving as tumor promoters and suppressors in a cancer type-specific manner (52).

In summary, we confirmed that these algorithm-specific features are extensively relevant to cancer types. Each method has a unique strength in a different aspect; therefore, all four methods must be incorporated for the comprehensive inference of cancer type classification.

### 4.2 Biological relevance of identified rules to cancer type classification

Besides essential features, several interesting classification rules were also obtained in this study (**Supplementary Table S4**). Here, some rules were examined. We found some features that can distinguish multiple cancer types with high impacts (passed counts >= 100). For example, the feature GO:0019002 (GMP binding) can classify BRCA, COADREAD, KIRC and OV, which is expected because GMP is the pharmacological target for treating multiple types of cancers (53–55). Another GO term group that can be used to classify multiple cancer type contains two GO terms, namely, GO:0031049 (programmed DNA elimination) and GO:0031052 (programmed DNA elimination by chromosome breakage), which are also involved in oncogenesis. Activation-induced deaminase is crucial in tumorigenesis because it is implicated in B cell lymphomas. DNA deaminases show preferred targeting, which provides solutions to identify their mutation foot-print in tumors. This finding also indicated their roles in genetic mutation in various cancer types (56). In addition to GO terms, some KEGG pathways can classify multiple cancer types, such as hsa00562 (Inositol phosphate metabolism - Homo sapiens (human)). We found it could be used to distinguish BRCA and COADREAD in this study. The main influential pathway contributing to CRC was inositol phosphate metabolism (57), which also had the most impact on the metabolic pathway in breast cancer. All these previous findings support our results and suggest the robustness of the methods in the present study.

## 5 Conclusion

This study was conducted on a cancer mutation dataset. After feature coding, irrelevant features were excluded using Boruta feature selection. Different feature ranking and IFS methods were then employed to identify the optimal number of features, construct efficient classifiers and extract interpretable classification rules. The results of the four methods were combined to identify the most important functional pathways and features, which were further discussed and validated with academic literature, providing a new understanding of the altered biological functions of different cancer types.

## Data availability statement

Publicly available datasets were analyzed in this study. This data can be found here. http://cbio.mskcc.org/cancergenomics/pancan_tcga/.

## Author contributions

ZZ, TH and Y-DC designed the study. JL and SD performed the experiments. JRL and JR analyzed the results. JL and JRL wrote the manuscript. All authors contributed to the article and approved the submitted version.

## Funding

This work was supported by the Shanghai Municipal Science and Technology Major Project (2017SHZDZX01), Strategic Priority Research Program of Chinese Academy of Sciences [XDB38050200, XDA26040304], the Fund of the Key Laboratory of Tissue Microenvironment and Tumor of Chinese Academy of Sciences [202002].

## Conflict of interest

The authors declare that the research was conducted in the absence of any commercial or financial relationships that could be construed as a potential conflict of interest.

## Publisher’s note

All claims expressed in this article are solely those of the authors and do not necessarily represent those of their affiliated organizations, or those of the publisher, the editors and the reviewers. Any product that may be evaluated in this article, or claim that may be made by its manufacturer, is not guaranteed or endorsed by the publisher.

## Supplementary material

Supplementary Table 1 Feature lists obtained by using four feature selection methods after Boruta feature filtering.

Supplementary Table 2 Detailed results for incremental feature selection under different feature lists and different classification algorithms.

Supplementary Table 3 Venn results for the feature sets used in the feasible SVM classifiers on different feature lists yielded by four feature selection methods.

Supplementary Table 4 Classification rules extracted by the optimal DT classifier under each feature list.
